# Timing-dependent effects of green tea supplementation and exercise intensity on oxidative stress in diabetic rats: a 2 × 2 × 2 factorial study

**DOI:** 10.3389/fspor.2026.1773906

**Published:** 2026-03-18

**Authors:** H. S. Muhammad Nurfatony, Oktia Woro Kasmini Handayani, Mashuri Eko Winarno, Bambang Priyono, Cahyo Yuwono, Heny Setyowati, M. Fadli Dongoran, Palmadi Putri Surya Negara

**Affiliations:** 1 Departement of Physical Education, Faculty of Sport Science, Universitas Negeri Semarang, Semarang, Indonesia; 2 Doctoral Program in Sport Education (Postgraduated), Universitas Negeri Semarang, Semarang, Indonesia; 3 Departement of Nutrition, Faculty of Medicine, Universitas Negeri Semarang, Semarang, Indonesia; 4 Departement of Physical Education, Faculty of Sport Science, Universitas Negeri Malang, Malang, Indonesia; 5 Department of Physical Education, Faculty of Teacher Training and Education, Universitas Musamus, Merauke, Indonesia; 6 Department of Agro-Industrial Technology, Faculty of Science and Technology, Universitas Darussalam Gontor, Ponorogo, Indonesia

**Keywords:** exercise intensity, exercise timing, green tea supplementation, oxidative stress, rat model, type 2 diabetes

## Abstract

Exercise and green tea supplementation have been shown to attenuate oxidative stress in type 2 diabetes; however, their interactive effects across different exercise timings and intensities have not been systematically investigated. This study aimed to examine the interactive effects of exercise timing, exercise intensity, and green tea supplementation on oxidative stress in a rat model of type 2 diabetes using a factorial experimental approach. Fifty male Wistar rats were randomly assigned to eight experimental groups (*n* = 5 per group) in a 2 × 2 × 2 factorial design according to green tea supplementation form (Traditional green tea infusion vs. standardized green tea extract), exercise intensity (low vs. moderate), and exercise timing (morning *light-phase* vs. evening *dark-phase*). The intervention was conducted over 8 weeks and involved treadmill-based exercise training combined with oral green tea supplementation. Serum malondialdehyde (MDA) was the sole oxidative stress biomarker assessed in this study. MDA serum levels were measured as an index of systemic oxidative stress. Three-way analysis of variance revealed a significant interaction among green tea supplementation form, exercise intensity, and exercise timing on serum MDA levels (*p* = 0.0019, *η*^2^ = 0.26). All intervention groups demonstrated significantly lower MDA levels compared with diabetic controls (*p* < 0.05). The combination of standardized green tea extract with moderate-intensity exercise performed in the morning (light-phase) produced the greatest reduction in MDA levels and showed a trend toward normalization when compared with normal controls. These findings suggest that green tea supplementation, exercise intensity, and exercise timing do not act independently but interact in a timing-dependent manner to modulate oxidative stress in diabetic rats. However, interpretation of the observed morning (light-phase) advantage should consider the nocturnal nature of rodents, which may limit direct translation to human chronobiological exercise recommendations. Further studies incorporating multiple oxidative and metabolic markers and human trials are warranted.

## 1 Introduction

Diabetes mellitus is a chronic metabolic disease with a rising prevalence globally. This condition is characterized by persistent hyperglycemia that triggers various cellular dysfunctions, including increased *reactive oxygen species* (ROS) and an imbalance in the endogenous antioxidant defense system (1). This imbalance contributes to oxidative stress, which is closely associated with the progression of diabetic complications, ranging from neuropathy to cardiometabolic dysfunction (2). A biomarker frequently used to assess lipid peroxidation is malondialdehyde (MDA), which reflects cell membrane damage induced by ROS (2, 3).

Various non-pharmacological strategies have been developed to address oxidative stress in diabetes, one of which is through nutritional interventions based on natural antioxidants (4). Green tea (Camellia sinensis) is rich in polyphenolic compounds, particularly catechins such as epigallocatechin gallate (EGCG), which have been associated with increased endogenous antioxidant activity and modulation of oxidative stress pathways in experimental models (5, 6). Previous experimental studies suggest that green tea extracts may improve total antioxidant capacity and lessen oxidative damage through enhanced scavenging of reactive oxygen species and upregulation of endogenous antioxidant defenses. However, the magnitude of these effects varies across models, possibly due to differences in extract composition, dosage, administration methods, and study duration (5). Therefore, considering the green tea dosage form such as traditional green tea infusion vs. standardized extract is important for achieving consistent biological effects in oxidative stress modulation.

In addition to nutritional interventions, physical exercise has also been shown to modulate oxidative stress and enhance endogenous antioxidant defenses and mitochondrial function in experimental models and clinical studies. Regular exercise can stimulate antioxidant enzyme activity and adaptive redox responses, contributing to improved oxidative balance. However, the relationship between exercise and oxidative stress is highly dependent on the intensity and duration of activity, with moderate intensity generally associated with beneficial adaptive responses and excessive intensity potentially exacerbating reactive oxygen species (ROS) production and oxidative damage. Review evidence indicates that moderate exercise enhances antioxidant defense capacity via hormetic mechanisms, whereas very high intensity or poorly tailored protocols may overwhelm the redox system and increase oxidative stress (7).

Another factor that has been suggested as a relevant factor (8). Circadian rhythms play an important role in regulating energy metabolism (9), hormonal responses, mitochondrial function, and the body's antioxidant system (10). Several studies indicate that physiological activity performed at different circadian phases may influence the body's metabolic and redox responses to exercise and supplementation, including in diabetic conditions, however, these effects appear to be context-dependent and influenced by species-specific activity patterns (3, 11). Nevertheless, studies systematically evaluating the effects of exercise timing on oxidative stress, particularly in combination with antioxidant supplementation, remain limited.

Overall, although there is evidence that green tea, aerobic exercise, and circadian rhythms each contribute to the regulation of oxidative stress, a limited number of studies have systematically assessed the effects of their interaction in diabetes models. This knowledge gap provides a foundation for evaluating whether a multimodal approach may provide synergistic effects compared to single interventions under specific experimental conditions.

Based on this background, this study aims to evaluate the effect of interactions between the form of green tea supplementation (traditional green tea infusion and standardized green tea extract), exercise intensity (low and moderate), and exercise timing (morning/light phase vs. evening/dark phase in rodents) on serum *malondialdehyde* (MDA) levels in a type 2 diabetes rat model. The main research question asked is whether these three factors interact simultaneously in modulating oxidative stress, compared to the effect of each factor separately.

The primary hypothesis of this study is that there is a three-way interaction between green tea supplementation, exercise intensity, and exercise timing on changes in MDA levels. Exploratory, it is assumed that the combination of green tea extract with moderate-intensity exercise performed at a specific biological time phase is expected to result in greater MDA reductions than other combinations. This hypothesis is designed to generate initial insights (hypothesis-generating) regarding the potential synergy between nutrition, exercise, and biological rhythms, not to confirm definitive physiological mechanisms.

## 2 Materials and methods

### 2.1 Research design

This study used a three-way factorial (2 × 2 × 2) experimental design that evaluated three main factors: (1) form of green tea supplementation (traditional green tea infusion vs. standardized green tea extract), (2) exercise intensity (low vs. moderate), and (3) exercise timing (morning/light phase vs. evening/dark phase in rodents), morning (light-phase) exercise sessions were conducted during the light (inactive) phase of the rat circadian cycle, whereas evening (dark-phase) exercise sessions were performed during the dark (active) phase. In addition, two control groups were added: healthy controls and diabetic controls without intervention, resulting in 10 treatment groups, each consisting of five rats (*n* = 5) (Table 1). Animals were randomly assigned to experimental groups; however, blinding was not implemented during the intervention period or outcome assessment. All procedures were carried out in accordance with ethical guidelines for the use of laboratory animals.

**Table 1:** Experimental design (2 × 2 × 2 factorial).

Exercise intensity	Exercise timing	Supplementation	Rats (*n*)
Low	Morning	Traditional	5
Low	Morning	Modern	5
Low	Evening	Traditional	5
Low	Evening	Modern	5
Moderate	Morning	Traditional	5
Moderate	Morning	Modern	5
Moderate	Evening	Traditional	5
Moderate	Evening	Modern	5
Control group			
NC (Normal Control)			5
DC (Diabetic Control)			5

Modern (standardized tea extract); traditional (traditional green tea infusion); morning (light-phase exercise 09:00–11:00); evening (dark-phase exercise 19:00–21:00).

### 2.2 Experimental animals

A total of 50 male Wistar rats (*rattus norvegicus*) (8–10 weeks old, weighing 200–250 g) were used in this study. The rats were obtained from an accredited laboratory and acclimatized for 7 days prior to the study, with environmental conditions including a room temperature of 22–25 °C, humidity of 50%–60%, a 12:12 h light-dark cycle, and access to food and water *ad libitum*.

The rats used must meet the inclusion criteria: healthy rats based on physical and behavioral examinations, free of injuries or mobility limitations, and with a body weight within the standard range of the strain or the standards used in the study. Rats will be excluded if they exhibit a post-induction hypoglycemic response, are physically injured, or are unable to perform all treadmill protocols.

### 2.3 Diabetes induction

Type 2 diabetes induction protocol by (12) using a combination of *Nicotinamide* (NA) and *Streptozotocin* (STZ). *Nicotinamide* injection at a dose of 95 mg/kg body weight was given intraperitoneally 15 min before *Streptozotocin* injection at a dose of 55 mg/kg body weight in citrate buffer pH 4.5.

Three days or 72 h after induction, the rats were tested for fasting blood glucose from the tail vein using a glucometer. Rats with levels ≥ 200 mg/dL were categorized as diabetic and included in the study.

Although the NA–STZ protocol successfully induced hyperglycemia, additional metabolic parameters such as insulin levels or insulin resistance indices were not assessed, which may limit the characterization of diabetes severity in this model.

### 2.4 VO_2_ max assessment and training protocol

#### 2.4.1 VO_2_ max test: exercise intensity determination

The stepwise treadmill test used in this study did not involve measuring respiratory gas exchange. The test aimed to determine maximal running speed as a basis for determining exercise intensity. The initial speed was set at a low level and gradually increased until the animal could no longer maintain the running speed despite mild stimulation. The maximal time obtained was then used to determine exercise intensity, which was 50% of maximal running time for the low-intensity group and 70% of maximal running time for the moderate-intensity group. This approach was chosen to ensure relative consistency of each individual's training load.

Exercise intensity was determined using a time-to-exhaustion treadmill protocol as a practical proxy for individual aerobic capacity. Although this approach allows for individualized workload adjustment, it does not directly quantify physiological markers such as VO₂ max or lactate threshold. The use of time-to-exhaustion as a proxy for exercise intensity represents an indirect approach and should be interpreted with caution when comparing results across studies employing direct VO₂-based measurements.

#### 2.4.2 Treadmill exercise training protocol

Exercise was performed 5 days per week for 8 weeks with an initial treadmill speed of 10 m/min, with a speed increase of 3 m/min every 2 min, and the treadmill at a constant incline of 10˚. The test was stopped when the rats were unable to maintain their position and showed signs of physiological fatigue. Exercise intensity was determined relative to these capacities: (1) Low intensity 50% of the rat's maximum running time, (2) Medium intensity 70% of the rat's maximum running capacity. Results were recorded as individual medical records to ensure appropriate workload adjustments for each animal.

#### 2.4.3 Exercise treatment time

Training sessions are conducted 5 days per week for 8 weeks, scheduled in the morning (light-phase) (09.00–11.00 am) or evening (dark-phase) (21.00–23.00 pm).

### 2.5 Green tea supplementation

Traditional green tea infusion. 10 grams of dried Camellia sinensis leaves were steeped in 100 mL of water at 90 °C for 25 min, then filtered and cooled to room temperature. Standardized green tea extract. 1 gram of commercial green tea extract (Herbilogy, Indonesia) was dissolved in 10 mL of water at 95 °C for 25 min, then filtered and cooled. Both preparations were administered orally via gavage at a daily dose of 1,040 mg/kg body weight according to the results of a study comparing the most optimal doses by (13), administered orally via gavage 15–20 min after exercise, during an 8-week intervention period.

Although the starting weight of the raw material differs between the traditional green tea infusion (10 g) and standardized green tea extract (1 g) preparations, both are adjusted to provide a daily dose equivalent to 1,040 mg/kg body weight. Both green tea preparations were administered at equivalent doses based on total dry mass; however, differences in catechin composition and bioavailability between traditional green tea infusion infusions and standardized extracts could not be quantified and may have influenced the observed effects.

The difference in grams reflects the difference in concentration of the bioactive compounds—the traditional green tea infusion leaf contains lower and more variable levels of catechins, while the standardized extract provides higher and more stable concentrations (14). The green tea supplementation dose of 1,040 mg/kg body weight refers to the total mass of the administered preparation, whether in the form of a traditional green tea infusion or a standardized extracts. Dose equivalency between the two preparations was estimated based on previous literature approaches that used dry matter mass as a reference, not on specific catechin or polyphenol content. This study did not perform chemical analysis to measure levels of catechin, *epigallocatechin gallate* (EGCG), or other bioactive compounds. Therefore, possible differences in composition, concentration, and bioavailability of active compounds between the preparations cannot be eliminated and is recognized as a methodological limitation.

### 2.6 Blood sampling

Blood samples were collected 24 h after the last exercise session to prevent acute training response effects. Blood was drawn via the retro-orbital vein under light anesthesia, then centrifuged at 3,000 rpm for 15 min to obtain serum.

### 2.7 MDA level measurement (ELISA)

Malondialdehyde (MDA) concentration was measured using a Rats MDA ELISA Kit (Bioassay Technology Laboratory, Cat. No. E0156Ra) based on Sandwich Immunoassay, with a sensitivity characteristic of 0.1 nmol/mL, Coefficient of Variation (CV) <10%, and absorbance reading of 450 nm. MDA blood examination using an ELISA Kit with general steps, namely serum diluted 1:5 with kit buffer, samples and standards were inserted into antibody-coated ELISA plates according to kit instructions, incubation, washing, and addition of enzyme conjugate were carried out according to the manufacturer's protocol, and absorbance was read using an ELISA microplate reader, then calculated using a standard curve of the 4-parameter Logistic Model (4PL) model.

Serum MDA levels were analyzed using a commercial ELISA kit manufactured for rat samples. This kit was used for rat serum samples, although cross-species specific validation was not explicitly stated by the manufacturer. Because no additional internal validation testing for rat species was performed, the use of this kit is recognized as a study limitation that could potentially impact the accuracy of the results. Nevertheless, all samples were analyzed uniformly under the same experimental conditions.

### 2.8 Research ethics statement

The protocol and all investigation procedures have been reviewed and approved by the Ethics Committee of the Faculty of Veterinary Medicine, Universitas Gadjah Mada, Yogyakarta, Indonesia with letter number: 62/EC-FKH/int./2025. The standards described in the ARRIVE (Animal Research: Reporting of *in vivo* Experiments) guidelines have been followed to address ethical aspects in animal research, to improve transparency and reproducibility of research and follow the 3Rs principle (Replacement, Reduction, Refinement) to ensure the welfare of laboratory animals.

### 2.9 Statistical analysis

A three-way factorial ANOVA was performed exclusively on the treatment groups in a 2 × 2 × 2 design (green tea form – exercise intensity – exercise time). Normal control (NC) and diabetic control (DC) groups were not included in the ANOVA model, but were used as external biological comparators to provide context for changes in MDA levels.

Statistical analysis was performed using SPSS version 25 and GraphPad Prism 10. The first stage included checking statistical assumptions using the Shapiro–Wilk normality test and the homogeneity of variance test using Levene's Test. Data were declared to meet parametric assumptions if the *p*-value > 0.05 for both tests.

Since the data met the parametric assumptions, the main analysis was performed using Three-Way ANOVA to evaluate the effect of green tea type (2 levels), exercise intensity (2 levels), and exercise time (2 levels) as well as the interaction between factors on serum MDA levels. Effect sizes were reported using eta-square (ƞ2) to estimate the contribution of variables to the total variation.

Further analyses were performed using the Tukey HSD test to identify differences between pairs of groups. A *p*-value <0.05 was considered significant in all analyses.

While the factorial design enabled assessment of interaction effects, no formal *a priori* power calculation was performed, and the relatively small sample size per group may have limited statistical power, particularly for detecting smaller interaction effects.

## 3 Results

### 3.1 Description statistics

Initial descriptive analysis was used to describe the pattern of MDA levels across all groups before conducting inferential testing. Overall, the results showed clear differences in MDA levels between groups. The healthy control group had the lowest MDA levels (Mean = 2.1 nmol/mL), while the diabetic control group experienced the highest increase (Mean = 7.8 nmol/mL), indicating successful induction of oxidative stress through *streptozotocin-nicotinamide*.

Both physical exercise and green tea supplementation reduced MDA levels compared to diabetic controls, but the reduction varied depending on the green tea dosage form, exercise intensity, and exercise time. The greatest reduction pattern was observed with the Modern-Moderate-Morning combination, which showed values approaching the normal physiological range. This suggests a possible synergistic effect between the intervention factors.

Table 2 descriptive statistical analysis shows that the highest MDA levels were found in the diabetes control group (7.8 ± 0.40 nmol/mL), while the normal control group showed the lowest value (2.1 ± 0.20 nmol/mL). Most intervention groups showed lower mean MDA levels with a consistent pattern, in which the use of standardized green tea extract, moderate-intensity exercise, and morning (light-phase) exercise time resulted in the lowest MDA levels (3.6 ± 0.21 nmol/mL), showing a trend toward normalization relative to non-diabetic controls.

**Table 2:** Mean ± SD value of MDA levels between treatment groups.

Group	Mean (nmol/mL)	SD
Normal Control (NC)	2.1	0.20
Diabetic Control (DC)	7.8	0.40
Traditional – Low – Morning	6.5	0.33
Traditional – Low – Night	6.9	0.31
Traditional – Moderate – Morning	5.2	0.29
Traditional – moderate – Night	5.6	0.27
Modern – Low – Morning	4.9	0.26
Modern – Low – Night	5.3	0.25
Modern – Moderate – Morning	**3**.**6**	**0**.**21**
Modern – Moderate – Night	4.1	0.23

Modern (standardized tea extract); traditional (traditional green tea infusion); morning (light-phase exercise 09:00–11:00); evening (dark-phase exercise 19:00–21:00).

Bold values indicate statistically significant differences (*p* < 0.05).

### 3.2 Statistical assumption test

Before the inferential analysis was carried out, the parametric assumption test data.

Table 3 shows that all groups exhibit a *p*-value > 0.05, indicating that the data are normally distributed. This indicates that the data distribution pattern is symmetrical and meets one of the important assumptions for using parametric analysis.

**Table 3:** Shapiro–Wilk normality test.

Group	Statistics W	*p*-value	Interpretation
Normal Control (NC)	0.96	0.482	Normal
Diabetic Control (DC)	0.94	0.331	Normal
Traditional – Low – Morning	0.95	0.410	Normal
Traditional – Low – Night	0.96	0.455	Normal
Traditional – Moderate - Morning	0.97	0.562	Normal
Traditional – Moderate – Night	0.96	0.499	Normal
Modern – Low – Morning	0.95	0.378	Normal
Modern – Low – Night	0.94	0.343	Normal
Modern – Moderate – Morning	0.97	0.601	Normal
Modern – Moderate - Night	0.96	0.472	Normal

Modern (standardized tea extract); traditional (traditional green tea infusion); morning (light-phase exercise 09:00–11:00); evening (dark-phase exercise 19:00–21:00).

The *p*-value in Levene's test is greater than *α* = 0.05, indicating that the variance between groups is homogeneous. Thus, the data meets the second assumption of parametric analysis and is suitable for proceeding to Three-Way ANOVA (Table 4).

**Table 4:** Results of the homogeneity of variance test (Levene's test).

Variables	Statistics F	*p*-value	Interpretation
MDA	1.87	0.142	Homogeneous

### 3.3 Analysis of variance (three-way ANOVA)

This analysis was used to determine the influence of each factor and the interactions between factors on MDA levels. The results showed an interdependent relationship between the treatment variables.

**Figure 1:**
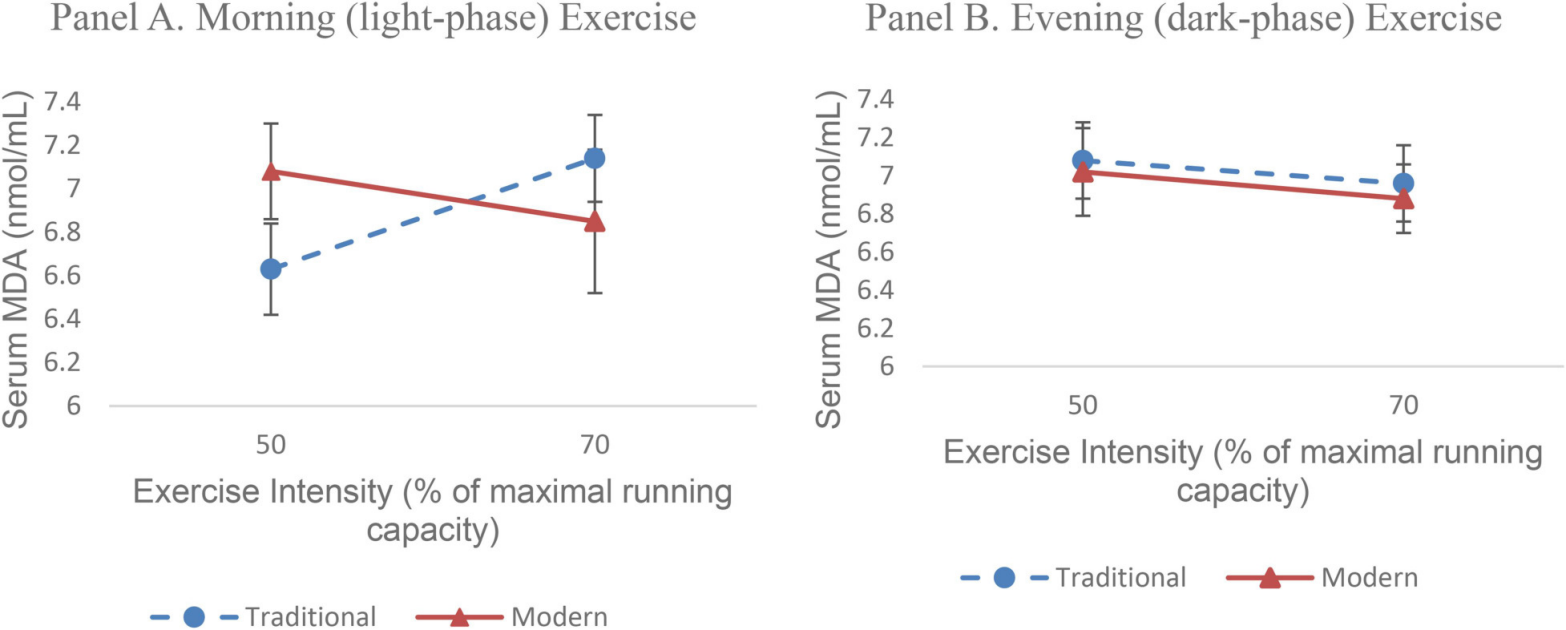
Three-way interaction of green tea supplementation, exercise intensity, and exercise timing on serum MDA levels in type 2 diabetic rats.

Figure 1 shown a visualization on the three-way interaction between the form of green tea supplementation (traditional green tea infusion and standardized green tea extract), exercise intensity (50% and 70% of maximum running capacity), and exercise time (morning *light-phase* and evening *dark-phase*) on serum *malondialdehyde* (MDA) levels in type 2 diabetic rats. Panel A represents the morning (light-phase) exercise condition, while Panel B represents the evening (dark-phase) exercise condition.

In both panels, the *X*-axis shows exercise intensity, while the *Y*-axis shows serum MDA levels (nmol/mL). Separate lines represent the types of green tea supplementation, with values presented as mean ± SEM.

Visually, the most consistent decrease in MDA was observed in the group receiving standardized green tea extract with moderate-intensity exercise (70%), particularly in the morning (light-phase) exercise group. This pattern differed from evening (dark-phase) exercise, where the decrease in MDA was relatively smaller at the same intensity and type of supplementation. This visualization supports the results of the Three-Way ANOVA analysis, which showed a significant interaction between the three factors on serum MDA levels.

Table 5 shows that three-way ANOVA analysis shows that tea extract, exercise intensity, and exercise timing each had a significant effect on MDA levels (*p* < 0.05). Furthermore, significant two-way interactions were found between tea extract and exercise intensity and between exercise intensity and exercise timing (*p* < 0.05). The interaction between tea extract and exercise timing did not reach statistical significance (*p* = 0.071), although it showed a trend toward a small to moderate effect size (ƞ^2^ = 0.06). Most notably, the three-way interaction between tea extract, exercise intensity, and exercise timing showed a highly significant effect with a large effect size (ƞ^2^ = 0.26), indicating that oxidative stress reduction is most optimally achieved through an integrated combination of interventions.

**Table 5:** Three-Way ANOVA results on MDA levels.

Factor	df	F	*p*-value	ƞ^2^
Tea Extraction	1	6.44	0.014	0.11
Exercise Intensity	1	9.32	0.003	0.15
Exercise Timing	1	4.85	0.029	0.08
Tea Extraction × Exercise Intensity	1	5.76	0.021	0.09
Tea Extraction × Exercise Timing	1	3.42	0.071	0.06
Exercise Intensity × Exercise Timing	1	4.11	0.047	0.07
Tea extraction × Exercise Intensity × Exercise Timing	**1**	**12.67**	**0.0019**	**0.26**

Bold values indicate statistically significant differences (*p* < 0.05).

### 3.4 Advanced analysis (tukey HSD)

*Post-hoc* analysis is needed to identify which groups differ significantly.

Table 6 shows that all intervention groups had significantly lower MDA levels compared to diabetic controls, with the greatest reduction observed in the standardized green tea extract group combined with moderate-intensity morning (light-phase) exercise (*p* < 0.001). Interestingly, MDA levels in this group were not significantly different from normal controls (*p*=0.067), indicating a trend toward normalization of oxidative stress to near-normal physiological levels.

**Table 6:** Tukey HSD pairwise comparisons results.

Group comparison	Mean difference	*p*-value
Diabetic vs. Normal	+5.7	<0.001
Diabetic vs. Traditional -Low-Morning	+1.3	0.041
Diabetic vs. Traditional -Moderate-Morning	+2.6	0.008
Diabetic vs. Modern-Low-Morning	+2.9	0.006
Diabetic vs. Modern-Moderate-Morning	**+4.2**	**<0.001**
Modern-Moderate-Morning vs. Normal	+1.5	0.067

Modern (standardized tea extract); traditional (traditional green tea infusion); morning (light-phase exercise 09:00–11:00); evening (dark-phase exercise 19:00–21:00).

Bold values indicate statistically significant differences (*p* < 0.05).

## 4 Discussion

Although this study demonstrated statistically significant variations in MDA levels between treatment combinations, it should be emphasized that MDA was the only biomarker measured. Therefore, these findings primarily reflect changes in lipid peroxidation and cannot be interpreted as a comprehensive representation of redox status, inflammation, or metabolic adaptation in general. The discussion regarding the involvement of molecular pathways such as Nrf2, NF-*κ*B, AMPK, circadian clock genes, and mitochondrial biogenesis is based on prior literature and was not directly assessed in this study. Because molecular signaling pathways such as Nrf2, AMPK, and circadian clock genes were not directly measured, mechanistic interpretations remain speculative and are intended as hypothesis-generating rather than confirmatory. In addition, body weight changes were not longitudinally monitored, which may represent a potential confounding factor influencing oxidative stress outcomes. Accordingly, the observed three-way interaction should not be interpreted as a basis for direct recommendations regarding precision exercise prescription or chrono-nutrition strategies in humans. Given the limited sample size, use of an animal model, and narrow scope of biomarkers, any translational implications should be considered with caution.

The findings of this study indicate that the combination of interventions in the form of green tea supplementation, exercise training, and exercise time has a significant effect on reducing malondialdehyde (MDA) levels in type 2 diabetic rats. The timing-dependent response biological response was found in the Standardized Green Tea Extract and Moderate Intensity and morning (light-phase) exercise group, which showed MDA levels approaching those of the normal control group. This pattern indicates a synergistic effect between intervention factors that work through antioxidant mechanisms, adaptation, and circadian rhythm modulation. However, this finding should be interpreted cautiously given the small sample size per group and the absence of additional oxidative or metabolic biomarkers.

The present findings indicate that the observed reduction in MDA is more closely associated with the underlying biological phase rather than clock-defined exercise timing. In nocturnal rodents, the light phase represents a period characterized by lower basal metabolic load, reduced endogenous reactive oxygen species (ROS) production, and a relatively stable redox environment. Exercise performed during this biological phase may therefore provide a controlled oxidative stimulus that enhances endogenous antioxidant defenses without exceeding redox buffering capacity. Importantly, this physiological condition may be functionally comparable to the early active phase in diurnal humans, such as the morning, when basal metabolic and oxidative stress levels remain relatively low. Accordingly, the timing-dependent effects observed in this study should be interpreted within a biological-phase framework rather than direct chronological equivalence.

### 4.1 Effectiveness of combination interventions compared to single interventions

The findings of this study confirm that the significant reduction in oxidative stress in type 2 diabetic rats cannot be explained by a single intervention in isolation, but rather is a combined result of exogenous antioxidant supply, adaptive stimulus from physical exercise, and synchronization of exercise timing with circadian rhythms. The results of the three-way interaction analysis showed that these three factors contributed simultaneously to reducing lipid peroxidation, as reflected in the decrease in MDA levels. In this finding in the context of diabetes physiology, metabolic dysfunction is usually multifactorial involving chronic oxidative stress, inflammation, insulin resistance, and biological rhythm disturbances, so that a single approach is often insufficient to produce meaningful improvement (15). Thus, this study supports an integrative framework in the oxidative stress management approach based on the integration of nutrition, exercise, and circadian rhythms. These findings are consistent with the literature suggesting that successful reduction of oxidative stress in chronic metabolic conditions often requires a multimodal approach rather than a single strategy (e.g., Exercise Physiology, Molecular Nutrition, and Experimental Endocrinology) (15, 16).

Integration of interventions as conducted in this study provides a broader response because it activates multiple molecular signaling pathways simultaneously. Green tea extract supplementation provides a major source of polyphenols such as EGCG which acts as a ROS scavenger and redox pathway modulator, moderate intensity exercise activates the PGC-1α, AMPK, and Nrf2 pathways (17) which increases endogenous antioxidant capacity, and adjusting exercise time to the physiological phase (light-phase exercise in the present rat model) increases metabolic efficiency through clock gene mechanisms and hormonal sensitivity that follows the circadian rhythm (18, 19), It should be noted that rats are nocturnal animals; therefore, exercise performed during the light phase represents the rest phase rather than the active phase, and the observed effects may reflect protocol-specific or stress-related responses. These three pathways do not operate independently, but rather mutually reinforce each other. Polyphenols help suppress initial ROS so that exercise does not produce excessive oxidative stress. Exercise helps increase the rate of polyphenol metabolism and cellular transport. Biological rhythms determine the time at which these two interventions produce the highest response (20). This synergy explains why the combination group (modern, moderate, morning) showed the most significant improvement.

### 4.2 The role of green tea supplementation: traditional green tea infusion vs. standardized green tea extract

The results of this study indicate that standardized green tea extract supplementation was associated with a greater reduction on reducing oxidative stress than traditional green tea infusion. This difference in biological response can be explained by variations in active compound content, polyphenol component bioavailability, and catechin stability in each dosage form (21). Green tea naturally contains *epigallocatechin gallate* (ECGC), *epicatechin* (EC), *epicatechin gallate* (ECG), and other flavonoids that act as powerful antioxidants (22). However, the concentration and stability of these compounds are highly dependent on the extraction method. traditional green tea infusion have limitations because the boiling process can cause thermal degradation of ECGC and a reduction in total polyphenol content. In contrast, standardized green tea extract are made using low-temperature standardization, solvent purification, and oxidative stabilization technologies, resulting in higher, more consistent bioactive compounds that are readily absorbed by the body (21).

Standardized polyphenol extracts have significantly higher ROS scavenging capacity than simple infusions, particularly in pathological conditions such as type 2 diabetes characterized by chronic oxidative stress. EGCG in the form of standardized green tea extract has been shown to increase cellular antioxidant capacity through the activation of the Nrf2-to-AP1 pathway, increasing the expression of SOD, CAT, and GPx enzymes (23), and inhibit proinflammatory pathways such as NF-KB (24). Previous studies also showed that EGCG was able to reduce MDA, 4-HNE levels (23), and other lipid peroxidation biomarkers, which makes green tea a relevant nutraceutical candidate in diabetes adjuvant therapy (25). The role of these bioactives is not only limited to scavenging free radicals, but also involves increasing mitochondrial activity, protecting cell membranes against oxidative damage, and increasing tissue redox.

Polyphenol supplementation works not only as a direct antioxidant, but as a modulator of signaling pathways (23). Type 2 diabetes involves decreased AMPK activity, increased mitochondrial ROS, and impaired lipid homeostasis. EGCG has been reported to increase AMPK phosphorylation, decrease ROS production through modulation of complexes I and III of the electron transport chain, and enhance mitochondrial biogenesis through activation of PGC-1*α* (26). These effects provide a physiological basis for why supplementation with standardized green tea extract containing ECGC at optimal doses can produce significant changes in oxidative stress profiles. Traditional green tea infusion with lower active ingredients may provide only minimal benefits, especially in pathological models that require higher doses to achieve therapeutic effects.

However, because catechin and EGCG concentrations were not directly quantified in this study, these mechanisms should be interpreted as plausible explanations based on prior literature rather than confirmed drivers of the observed effects.

### 4.3 Exercise intensity: mechanisms of hormesis and mitochondrial adaptation

Moderate intensity exercise has a more significant effect on reducing MDA levels than low intensity exercise (27). This finding is in line with the concept of exercise-induced hormesis (28), namely that exposure to moderate amounts of physiological stress can have positive adaptive effects that strengthen the cellular defense system. In type 2 diabetes, an imbalance between free radical production and clearance is often accompanied by impaired mitochondrial function and decreased endogenous antioxidant capacity (29). Therefore, moderate-intensity exercise appears to be in an timing-dependent response zone that is intense enough to stimulate mitochondrial adaptation and improvement in redox capacity, but not so intense that it produces excessive ROS and exacerbates oxidative stress.

Moderate intensity exercise increases the expression of the transcription factor PGC-1*α*, which acts as a key regulator of mitochondrial biogenesis (28). PGC-1*α* activation further stimulates an increase in the number and quality of new mitochondria, including increased activity of electron transport chain (ETC) complexes, thereby improving the efficiency of ATP production and reducing electron leakage, which is a major source of ROS (30). AMPK pathway activity, which occurs frequently during moderate-intensity exercise, also plays an important role in the activation of PGC-1*α* (31) and increased ability of cells to maintain energy homeostasis (32). Thus, adaptations at the mitochondrial level induced by moderate-intensity exercise provide a strong mechanistic framework for the reduction in MDA in this study. Moderate-intensity exercise has been shown to increase the activity of endogenous antioxidant enzymes (33) such as *superoxide dismutase* (SOD) (34), *catalase* (CAT) (35), and *glutathione peroxidase* (GPx) in muscle and liver tissue (7). In diabetic models, increased activity of these enzymes is essential to offset the high ROS production resulting from chronic hyperglycemia.

Overall, this study confirms that moderate-intensity exercise is an essential component of any combination intervention. This intensity provides an optimal balance between adaptive stimulus and physiological load, maximizing antioxidant responses and improving mitochondrial function. When combined with green tea supplementation and appropriate training timing, moderate-intensity exercise produces greater biological synergy than any single intervention, making it the timing-dependent response option for reducing oxidative stress in a model of type 2 diabetes.

### 4.4 Exercise timing and circadian rhythms: the importance of chrono-exercise in modulating oxidative stress

Experimental studies in diabetic model rats with a fat diet plus STZ, aerobic exercise performed in the light phase and dark phase showed differences in the expression of circadian clock proteins (e.g., BMAL1, PER2) as well as mitochondrial dynamics regulatory proteins such as MFN2 and DRP1, exercise in the early light phase (ZT3) and dark phase (ZT15) improved skeletal muscle clock dysfunction due to diabetes, but the most consistent improvement in the dark phase (ZT15) showed that tissue sensitivity to exercise is time-related (36). These findings suggest that exercise timing influences metabolic and redox responses; however, in nocturnal animals such as rats, the biological interpretation of light-phase (morning) exercise requires careful consideration.

Modern chronobiology literature suggests that mitochondrial function and cellular redox capacity are influenced by the 24-hour cycle of clock gene expression modulating glucose metabolism, the NAD^+^ or SIRT1 pathway, and endogenous ROS production (37). Thus, training at a time appropriate to the biologically active phase allows synchronization between the training signal and peak endogenous antioxidant activity, resulting in a more stable redox state and resistance to lipid peroxidation.

In diurnal human populations, morning training tends to provide a more stable biochemical profile with generally lower levels of oxidative stress, hormones, and inflammatory parameters compared to afternoon or evening training in both athlete and non-athlete populations (38). The results of this study support the view that in conditions of chronic oxidative stress such as diabetes, aligning exercise time with biological rhythms (*chrono-exercise*) has better adaptation potential than exercising at any time.

A chrono-exercise strategy (exercising during the optimal biological phase) can enhance antioxidant effects, suppress lipid peroxidation, and minimize the impact of ROS, potentially preventing long-term oxidative complications. This approach aligns with the trend in translational research that emphasizes individualizing lifestyle interventions based on an individual's chronotype, sleep-wake cycle, and biological rhythms (39). These time-based interventions can also be utilized in metabolic rehabilitation, diabetes complication prevention, and public health strategies. For example, for T2DM patients who regularly consume nutraceutical supplements such as polyphenols, combining them with a morning (light-phase) exercise schedule and a circadian-friendly diet could form a precision medicine-based intervention package.

It is important to emphasize that rats are nocturnal animals; therefore, the morning (light-phase) exercise condition in this study corresponds to the light (inactive) phase of the circadian cycle, whereas the evening (dark-phase) exercise condition represents the dark (active) phase. Accordingly, the observed reduction in MDA levels following morning (light-phase) exercise should be interpreted as a protocol-specific and timing-dependent response, rather than as evidence that light-phase exercise is biologically optimal for nocturnal species. These findings therefore warrant cautious interpretation with respect to translational relevance, as the temporal alignment of exercise relative to the active–rest cycle differs fundamentally between nocturnal rodents and diurnal humans.

### 4.5 Synergistic mechanisms: nutrition-exercise-circadian integration

Moderate-intensity exercise has long been known to increase the activity of the endogenous antioxidant system and mitochondrial biogenesis. Studies in diabetic rats have shown that aerobic exercise, especially when combined with appropriate circadian rhythms, restores mitochondrial dynamic function and balances ROS levels and cellular energy (36). Meanwhile, the cellular redox system is influenced by antioxidant regulatory pathways, such as NRF2, a master transcription factor that initiates the expression of antioxidant enzymes when triggered by oxidative stress or physiological stimuli. Studies in rats lungs have shown that NRF2 expression oscillates in a 24-hour cycle, suggesting that the endogenous antioxidant system is controlled by circadian rhythms (38, 40). Thus, polyphenol supplementation (as exogenous antioxidant donors) together with exercise (redox and energy stimulation) can enhance NRF2 activation and cellular defense pathways, while mitochondrial biogenesis helps improve oxidative efficiency and reduce electron leakage (30). This combination creates a redox-optimized mitochondrial state, meaning the mitochondria are efficient and the cells are able to handle high oxidative loads.

Recent literature emphasizes that the interaction between the biological clock system and the redox system is very important, the core of the circadian clock influences the regulation of redox pathways, while oxidative stress or metabolic changes can affect the expression of clock genes (41). By exercising at a time aligned with daily rhythms, such as during the biologically appropriate active phase for a given species, the training stimulus and antioxidant supply from green tea can peak during periods of high metabolic activity, allowing antioxidant hormones and enzymes to be at their optimal phase. This allows for more efficient adaptation. This combination suggests that the benefits of exercise and nutrition depend not only on dosage but also on the alignment of biological time—a concept called chrono-exercise or chrono-nutrition (42).

### 4.6 Counter-arguments and justification

Some potential criticisms may arise, such as the possibility that the decrease in MDA may be due to spontaneous adaptation, not intervention. However, the diabetic control group maintained high MDA levels, suggesting that spontaneous adaptation does not occur without intervention. Circadian effects may not be significant in nocturnal animals. Although rats are nocturnal, circadian studies show that responses to exercise timing differ between early and late active phases. The data from this study demonstrate a significant difference between morning (light-phase) and evening (dark-phase) exercise. The differences between groups may be driven by individual biological variation. The variance between groups was homogeneous (Levene's Test *p* = 0.142), suggesting that biological variation is not the primary source of the differences. The findings of this study remain consistent and statistically valid.

### 4.7 Translational implications for human clinical studies

Although this study was conducted in a rats model of type 2 diabetes, the observed physiological patterns provide a strong basis for translational implications, such as concentrated green tea extract has the potential to be a nutraceutical strategy in the management of diabetic oxidative stress, moderate-intensity exercise consistently shows antioxidant benefits, morning (light-phase) exercise time is potentially associated with lower MDA time for patients to achieve the greatest metabolic benefits, and a multi-model approach is more effective than a single intervention. However, further validation through human clinical trials is needed, particularly regarding extract dosage, exercise intensity, and synchronizing exercise with human circadian rhythms.

## 5 Conclusion

This study demonstrates that oxidative stress responses, as reflected by serum malondialdehyde levels, are influenced by the interactive effects of green tea supplementation, exercise intensity, and exercise timing in a rat model of type 2 diabetes. The significant three-way interaction observed in this study indicates that these factors do not act independently, but rather modulate oxidative stress in a timing-dependent manner when combined.

Among the tested conditions, the combination of standardized green tea extract, moderate-intensity exercise, and morning (light-phase) exercise timing was associated with lower serum MDA levels compared with other treatment combinations and the diabetic control group. It is important to note that rats are nocturnal animals; therefore, “morning (light-phase) exercise” in this experimental context corresponds to the light (rest) phase rather than the biologically active phase typical of humans. These findings suggest that both the form of green tea supplementation and the temporal context of exercise may influence oxidative stress regulation in diabetes, although the underlying mechanisms were not directly assessed in the present study.

From a conceptual perspective, the results support the view that oxidative stress regulation may involve interactions between nutritional factors, exercise-induced adaptations, and biological timing. However, these interpretations remain speculative and should be considered within the limitations of the current experimental design.

This study is limited by the use of a single oxidative stress biomarker, a small sample size per group, and an animal model, which restricts direct translation to human populations. Accordingly, the timing-dependent effects observed in this study should be regarded as model-specific and cannot be directly extrapolated to humans, whose circadian organization and activity patterns differ fundamentally from those of rodents. The present findings should therefore be regarded as preliminary and hypothesis-generating. Future studies incorporating additional redox and inflammatory markers, molecular signaling analyses, and clinical populations are warranted to further clarify the role of exercise timing and nutritional interventions in the management of type 2 diabetes.

Because rats are nocturnal animals, the morning (light-phase) exercise condition examined in this study corresponds to the light (rest) phase and should not be directly extrapolated to clock-based exercise recommendations for humans, who exhibit a diurnal circadian pattern; rather, these findings highlight the importance of biological-phase–dependent mechanisms, whereby exercise performed under low basal metabolic and oxidative stress conditions may optimize endogenous antioxidant responses in both species.

## Data Availability

The raw data supporting the conclusions of this article will be made available by the authors, without undue reservation.
